# The Epilepsy–Aphasia Syndrome Gene, Cnksr2, Plays a Critical Role in the Anterior Cingulate Cortex Mediating Vocal Communication

**DOI:** 10.1523/ENEURO.0532-24.2024

**Published:** 2025-01-16

**Authors:** Kazi Hossain, Eda Erata, Lucio Schiapparelli, Scott H. Soderling

**Affiliations:** ^1^Departments of Cell Biology, Duke University Medical School, Durham, North Carolina 27710; ^2^Neurobiology, Duke University Medical School, Durham, North Carolina 27710

**Keywords:** aphasia, Cnksr2, epilepsy, USVs

## Abstract

Epilepsy–aphasia syndrome (EAS) is a spectrum of childhood disorders that exhibit complex comorbidities that include epilepsy and the emergence of cognitive and language disorders. CNKSR2 is an X-linked gene in which mutations are linked to EAS. We previously demonstrated Cnksr2 knock-out (KO) mice model key phenotypes of EAS analogous to those present in clinical patients with mutations in the gene. Cnksr2 KO mice have increased seizures, impaired learning and memory, increased levels of anxiety, and loss of ultrasonic vocalizations (USVs). The intricate interplay between these diverse phenotypes at the brain regional and cell-type level remains unknown. Here, we leverage conditional deletion of the X-linked Cnksr2 in a neuronal cell-type manner in male mice to demonstrate that anxiety and impaired USVs track with its loss from excitatory neurons. Finally, we further narrow the essential role of Cnksr2 loss in USV deficits to excitatory neurons of the ACC, a region in mice recently implicated in USV production associated with specific emotional states or social contexts, such as mating calls, distress calls, or social bonding signals. Together, our results reveal Cnksr2-based mechanisms that underlie USV impairments that suggest communication impairments can be dissociated from seizures or anxiety. Furthermore, we highlight the cortical circuitry important for initiating USVs.

## Significance Statement

Epilepsy–aphasia syndromes are at the severe end of a spectrum of cognitive–behavioral symptoms that are seen in childhood epilepsies and are currently an inadequately understood disorder. The prognosis of Epilepsy–aphasia syndrome is frequently poor and patients have life-long language and cognitive disturbances. We show that the deletion of Cnksr2 specifically within glutamatergic neurons of the anterior cingulate cortex leads to ultrasonic vocalization impairments, providing an important new understanding of the modulation of vocal communication.

## Introduction

Childhood and infancy epilepsy are complex, debilitating disorders with largely unknown causes. These conditions encompass a spectrum, ranging from epilepsy to epilepsy–aphasia syndrome (EAS), predominantly affecting males. At the severe end of this spectrum lie Landau–Kleffner syndrome and continuous spike and wave during slow-wave sleep syndrome. These syndromes are characterized by seizures, electrical status epilepticus during sleep, cognitive disability, and aphasia (Deonna, 1991; Tassinari et al., 2000; Wirrell et al., 2012). The treatment options for EAS are limited, with most children receiving speech therapy and anticonvulsants for seizures. However, these treatments often fall short in addressing cognitive impairments, resulting in many patients enduring persistent language and cognitive challenges (Dugas et al., 1976; Moreno-Flagge, 2013).

Recent studies have implicated heterozygous loss-of-function mutations of CNKSR2 with EAS, which are characterized by seizures, aphasia or the progressive loss of language, attention deficits, and X-linked neurodevelopmental disorders (Polla et al., 2019; Bonardi et al., 2020; Higa et al., 2021; Kang et al., 2021). The CNKSR2 gene encodes the connector enhancer of the kinase suppressor of Ras 2, a scaffolding protein that may influence the mitogen-activated protein kinase pathways downstream of Ras (Ito et al., 2021; Ito and Nagata, 2022). This protein, comprising 1,034 amino acids, contains various protein and membrane binding domains such as PDZ, sterile alpha motif, pleckstrin homology (PH), conserved region in connector of kinase, and C-terminal PDZ-binding motifs. CNKSR2 may also link signal transduction to membrane and cytoskeletal remodeling (Ito et al., 2021; Ito and Nagata, 2022). Notably, its location on the X chromosome fits with the male predominance in EAS patients (Higa et al., 2021). We previously developed a conditional Cnksr2 knock-out mouse model, whose phenotype, when ubiquitously deleted, successfully models aspects of primary human patient phenotypes in adult male mice (Erata et al., 2021). This conditional knock-out (cKO) model of Cnksr2 loss offers a unique opportunity to further investigate the basis of the diverse phenotypes that model EAS symptoms observed in human patients.

A key, but enigmatic phenotype of EAS associated with CNKSR2 mutations, is the progressive loss of vocal communication in children with this disorder. Although there are clear differences between human language and mouse vocal communication, loss of ultrasonic vocalizations (USVs) was observed in the Cnksr2 KO mice. However, the genetic-to-circuit mechanisms of this pathology were unknown. Recent debates have emerged about the cortex's role in USV production in mice (Arriaga and Jarvis, 2013). Damage to the M1 region of the motor cortex was found to slightly alter the USV frequency without affecting syllable composition or amplitude (Okobi et al., 2019). Conversely, Hammerschmidt et al. (2015) found that genetically removing all excitatory neurons in the cerebral cortex did not impede USV production, as cortex-lacking mice produced USVs comparable to control mice. However, other mammalian studies highlight the importance of cortical circuits in vocalizations, especially the ACC (Bennett et al., 2019). Vocal behaviors, which vary in complexity, involve three key components: respiratory movements, laryngeal activity, and supralaryngeal activity (Beckstead et al., 1980; Fort et al., 1994; Ezure et al., 1998). The motor neurons controlling these activities are in the ventral horn of the spinal cord, as well as in the pons and medulla. The coordination of various motor neurons is managed by a complex network that includes the lateral pontine reticular formation, the ventrolateral parabrachial area, the anterolateral and caudal medullary reticular formation, and the nucleus retroambiguus (Slugg and Light, 1994). This network is directly connected to the phonatory motor neurons and receives sensory input from laryngeal, pulmonary, and oral mechanoreceptors through the solitary tract nucleus and the principal and spinal trigeminal nuclei (Jürgens, 2002). Vocalization production requires inputs from the periaqueductal gray (PAG) in the midbrain and the adjacent lateral tegmentum (Jürgens, 2009). Control over vocalization initiation or suppression involves two cortical regions: the medial prefrontal cortex (mPFC) and the anterior cingulate cortex (ACC; Bennett et al., 2019). These circuits connect to the PAG, then to the reticular formation in the pons, and finally to the phonatory motor neurons (Simonyan, 2014). This vocalization circuit is conserved across humans, nonhuman primates, and rodents, including rats. In rats, the mPFC and ACC have been extensively studied for their roles in controlling ultrasonic vocalizations (USV; Bennett et al., 2019). In mice, USVs elicited during social interactions are high-pitched frequencies (30 kHz) used in mating or to attract attention for nursing needs for adult males and neonatal pups, respectively (Branchi et al., 2001). Recently, in mice, it was discovered that the ACC is involved in USV production (Gan-Or and London, 2023) and thus may be a prime candidate for further analysis regarding EAS.

Beyond the question of circuitry dysfunction that may underlie EAS, it is also unclear whether the associated seizures, which often precede aphasia, contribute to vocal communicative deficits (Marques Mendes et al., 2018). There are also other epilepsy/seizure-related disorders in which patients also have language deficits. Some examples are Koolen–de Vries syndrome (KdVS) and FOXP1 syndrome (Koene et al., 2023; Shalev et al., 2023). Patients with KdVS have a 100% likelihood of developing language disorders and a 33% likelihood of acquiring seizures (Karamik et al., 2023; Khan et al., 2022). The diagnosis of KdVS was recently established in a proband who has either a heterozygous 500–650 kb deletion at chromosome 17q21.31 that includes *KANSL1* or a heterozygous intragenic pathogenic variant in *KANSL1*, suggesting mutations of this gene may underlie the disorder (Karamik et al., 2023). FOXP1 syndrome is also characterized by delays in early motor and language milestones, mild-to-severe intellectual deficits, speech and language impairments, and epilepsy (Benvenuto et al., 2023). However, there are many other instances of disorders in which epilepsy/seizures are not associated with language loss as well as cases where language loss is not associated with epilepsy, such as Smith–Magenis syndrome in which patients are diagnosed with language disorders but lack seizures (Korteling et al., 2023). Thus, it remains unclear whether the seizures associated with CNKSR2 mutations are functionally linked to the subsequent language loss.

Here, we report alterations in neural activity in the absence of Cnksr2 for both excitatory and inhibitory neurons in vitro and in vivo. We also find, using conditional *Cnksr2* knock-out mice, that the vocal communication phenotypes of the KO mice can be dissociated by deletion of *Cnksr2* in either GABAergic or glutamatergic neurons. Deletion of *Cnksr2* in glutamatergic neurons leads to elevated levels of anxiety and loss of ultrasonic vocalizations in adult male mice, while deletion in GABAergic neurons does not. Further analysis also reveals that Cnksr2 expression in glutamatergic neurons within the ACC is important for USV production. Taken together, these results provide insight into the molecular functions of Cnksr2 and how its loss in distinct cell types leads to the diverse phenotypes associated with EAS.

## Materials and methods

### Stereotaxic injections

Mice were injected with AAV2/9 viruses sourced from Addgene, featuring a viral titer of 2.3 × 10^13^. To target the medial prefrontal cortex, we used coordinates of +1.5 mm anterior of bregma, 1.5 mm ventral, and 0.5 mm lateral from the midline. For targeting the anterior cingulate cortex, the coordinates were +1.0 mm anterior of bregma, 0.5 mm ventral, and 0.5 mm lateral from the midline. The injections were performed on postnatal day 30, using either AAV2/9-*CamKII-Cre-GFP* or AAV2/9-*CAG-tdTomato*. During the surgical procedure, the mice were anesthetized with isoflurane, using 3% for induction and maintaining 1–2% throughout. The injections were administered with micropipettes using a Nanoject, with each hemisphere receiving a volume of 90 nl. The rate of injection was maintained at 9 nl every 30 s. The needle was carefully lowered at a rate of 0.2 mm every 30 s from the dura. After each injection, we allowed 5 min for the virus to settle in the brain before retracting the needle by 0.2 mm. An additional 30 s was then given to ensure further settling of the virus, after which the needle was completely removed from the brain. Postoperative care included treatment with meloxicam and bupivacaine, and the mice were closely monitored 24 and 48 h after the surgery.

### Virus preparation

Php.eB vector was obtained from Chan et al. (2017). Php.eB-CAG-tdTomato and PhP.eB-*CamKII-Cre-GFP* were generated using HEK-293T cells. The process commenced when the HEK cells reached approximately 70% confluency, using 12 plates for each virus. The transfection involved 15 µg of the viral serotype, 15 µg each of Php.eB-CAG-tdTomato and PhP.eB-*CamKII-Cre-GFP* DNA vectors, and 30 µg of the helper virus pAD delta f6. The DNA mixture was combined with OPTI-Prep and added dropwise to the HEK cells. Approximately, 6 h post-transfection, the media of the HEK cells was changed. The cells were then harvested and processed for virus purification 3 d after the transfection. For viral purification, the harvested HEK-293T cells were lysed, and the virus was concentrated using an OPTI-prep density gradient (Sigma-Aldrich, #D1556). Following this, the newly obtained virus was titered to assess the yield and ensure it was within acceptable limits.

### Multielectrode array

In our study, we utilized a multielectrode array (MEA) system. We plated postnatal day 0 neurons from the *Cnksr2^fl/y^;Emx-Cre^−/−^* or *Cnksr2^fl/y^;Emx-Cre^+/−^* cortex in a 48-well MEA plate (Lumos 48, Axion Biosystems). Neuronal activity was recorded on Days 7, 10, and 13 after plating [day in vitro (DIV)]. Each well of the MEA plate contains a 4 × 4 grid of electrodes, each with a diameter of 50 nm and an electrode spacing of 350 mm from pole to pole. The wells were coated with 1 mg/ml of poly-ʟ-lysine in sodium borate buffer at pH 8.5. Neurons were plated at a density of 50,000 cells/5 µl in each well, specifically targeted onto the electrode grid within the inner well. One and a half hours after plating, 300 µl of growth media was added to the neurons. The growth media was changed at 5 DIV, and subsequently each time the neurons were recorded.

Recordings of local field potentials were conducted at a temperature of 37°C and 5% CO_2_, using a Maestro MEA system and AxIS software (Axion Biosystems). Ten minutes after placing the MEA plates on the stage, recordings of 10 min duration were taken for calculating the metrics. For data analysis, only wells with 14 out of 16 active electrodes were considered. Data acquisition was done at a rate of 12.5 kHz, with a digital Butterworth bandpass filter set between 200 and 3,000 Hz. The threshold for spike detection was fixed at six standard deviations. Independent measurements were taken from triplicate MEA plates, with each condition covering 16–24 wells. Electrode bursting was defined as a minimum of five spikes, each separated by <100 ms. Representative raster plots were generated using the Neural Metrics Tool (Axion Biosystems). Data collection was performed using three different MEA plates.

### Elevated zero maze

In our study, mice were evaluated for behaviors indicative of anxiety using the raised zero maze. Each mouse was placed in a sheltered section of the maze, where it was given 5 min for uninhibited exploration. This was conducted under subdued lighting conditions, maintained at 40–60 lux. To record their movements, a high-definition camera was positioned 180 cm above the center of the maze. The recorded movements were subsequently analyzed using EthoVision XT7 software from Noldus Information Technology. The EthoVision XT7 software generated tracking patterns, which yielded various data points. These included the duration the mice spent in open zones, the total distance they covered, their speed in centimeters per second, and the total count of movements they made through open spaces between the two enclosed sections of the maze. This comprehensive analysis allowed for a detailed assessment of behaviors related to anxiety in the mice.

### Adult USVs

Prior to the experimental examination, the animals were housed separately for a week. We selected *C57/B6J* female mice to serve as interaction partners. To prime the male mice, we exposed them to used bedding from the *C57/B6J* female partners, followed by a cage change on the subsequent day. This priming routine was repeated twice. Before the actual testing, the male mice were acclimatized to the recording spaces for 10–15 min daily. On the test day, we visually checked the estrus phase of the *C57/B6J* females, including only those females in estrus in the experiment. The testing procedure began by placing the male mice in the test area for 2 min Then, a *C57/B6J* female in estrus was introduced, allowing the mice to interact for 8 min. During the entire 10 min duration of the experiment, ultrasonic vocalizations emitted by the mice were captured as audio waveforms. These recordings were later analyzed using Avisoft-SASLab Pro software. This process ensured a controlled environment for observing and recording the interactions and vocalizations, facilitating precise and detailed behavioral analysis.

### Retro-orbital injections

In our study, *Cnksr2^fl/y^* mice were injected with 2 µl of PhP.eB virus into their eye socket to facilitate viral spread through the ventricles. The injections were carried out under anesthesia. During this procedure, the male mice were head-fixed and mounted on a stereotaxic rig. For the control and experimental groups, PhP.eB-*CAG-tdTomato* and PhP.eB-*CamKII-Cre-GFP* viruses were used, respectively. After the injections, the mice were closely monitored for any immediate postinjection effects. This monitoring took place at two key time points: 24 and 48 h postinjection. This careful observation helped ensure the well-being of the mice and the successful administration of the viral injections.

### EEG recording

The EEG and EMG data were sampled at a rate of 500 Hz, employing a high-pass filter at 1.0 Hz and a low-pass filter at 45 Hz. EEG1, EEG2, and EMG signals were collected using Sirena Acquisition software from Pinnacle Technologies. Both EEG1 and EEG2 channels were differential recordings sharing a common reference electrode. To detect epileptiform discharges, we used Seizure Pro software (Pinnacle Technologies). Spike–wave complexes that met specific criteria—frequency between 2 and 10 Hz, voltage twice the background EEG, and a minimum duration of 1 s—were included in the quantification of epileptiform discharges. For identifying electrographic seizures, we applied an automated line-length search method to all EEG channels. The threshold was set at a line length of 5,000 per second, using a 10 s search window and a 1 s sliding window. High-amplitude events exceeding this threshold, containing more than one spike-like event per second and lasting longer than 10 s, were classified as electrographic seizures. Events detected by the Seizure Pro software underwent confirmation or exclusion through visual inspection of both EEGs and video recordings. Nonrapid eye movement sleep (NREMS) periods were identified by extracting delta frequency (1–4 Hz) from FFT-transformed EEG data. These data were visually inspected and manually scored. Based on delta power, three states were delineated: wake/rapid eye movement sleep (REMS), NREMS, and transition periods between NREMS and wake/REMS. This comprehensive approach allowed for a detailed and accurate analysis of the EEG/EMG data in relation to the animals' behavior and neurological state.

### Olfactory test

Olfactory testing was run with mice that had been housed individually for at least a week as described (Rodriguiz et al., 2004). Briefly, animals were compared for their abilities to discriminate the odors from urine from estrus female versus saline. Fifty microliters of urine was pipetted onto 1 cm × 1 cm cotton wadding and was placed into Tissue-Tek cassettes (3 cm × 4 cm × 0.4 cm; VWR Scientific) mounted on opposite walls of the home cage, 1 cm above the floor. The time spent with each cassette was coded for 5 min using the Noldus Observer (Noldus Information Technology, Leesburg, VA). Selection was defined as the mouse sniffing or manipulating the cassette or being within 1 body length of the cassette and oriented toward it. The preference score for the female urine odor was calculated as the time spent with the urine cassette relative to the total time spent in both cassettes (urine plus saline).

#### Parallel reaction monitoring (PRM) targeted proteomics

For the PRM analysis, 28 µg of total protein from microdissected ACC and mPFC samples was used. Cortical brain region samples were supplemented with SDS to a final concentration of 5% for digestion. Samples were then reduced with 10 mM dithiothreitol for 30 min at 80°C and alkylated with 20 mM iodoacetamide for 30 min at room temperature. Next, they were supplemented with a final concentration of 1.2% phosphoric acid and 1,440 ml of S-Trap (ProtiFi) binding buffer (90% MeOH/100 mM TEAB). Proteins were trapped on the S-Trap, digested using 20 ng/ml sequencing grade trypsin (Promega) for 1 h at 47°C, and eluted using 50 mM TEAB, followed by 0.2% FA, and lastly using 50% acetonitrile (ACN)/0.2% FA. All samples were then lyophilized to dryness and resuspended in 15 ml 1%TFA/2% ACN containing 12.5 fmol/ml yeast alcohol dehydrogenase (ADH_YEAST). A sample pool quality control was created by taking 5 ml from each sample, which was run periodically throughout the acquisition period.

#### Targeted liquid chromatography-PRM

Liquid chromatography-PRM (LC-PRM) was performed using an Evosep One UPLC coupled to a Thermo Orbitrap Astral high-resolution accurate mass tandem mass spectrometer (Thermo Fisher Scientific). Briefly, each sample loaded Evotip was eluted onto a 1.5 µm Evosep 150 µm ID × 15 cm performance (Evosep) column using the SPD30 gradient at 55°C. Data collection on the Orbitrap Astral mass spectrometer was performed in a targeted PRM mode of acquisition set to target three unique peptides for CNKSR2 and three unique peptides for each of the following normalizing proteins; PLEC, BSN, and MYH10. The instrument was set to acquire a precursor MS scan in the Orbitrap from *m/z* 325–1,400 at *R* = 120,000 [target automatic gain control (AGC), 500%; max injection time (IT), 50 ms] with PRM spectra acquired in the Astral (target AGC, 100%; max IT, 20 ms). For all experiments, HCD energy settings were 27%. Raw LC-PRM data files were processed in Skyline (v24.1.1; MacCoss Lab, University of Washington) with manual integration and selection of at least three product ions per targeted precursor ion. The raw area under the curve data were exported for each targeted precursor, and all precursors to the same protein were summed prior to any relative comparisons across biological conditions. To account for subtle differences in sample loading, the average summed signal from three additional proteins was used to normalize CNKSR2 levels prior to relative comparative analysis.

### Statistical analysis

Information on experimental repetitions and the statistical methods used is detailed in the captions of each figure. All studies were conducted at least three times. Statistical evaluations and graph creation were done using GraphPad Prism or Microsoft Excel. The Shapiro–Wilk test checked data for normal distribution to decide between parametric and nonparametric statistical tests. When multiple comparisons were made, suitable post hoc tests were applied, as noted in the figure captions. All graphed data are presented as mean ± SEM.

## Results

### Multielectrode array assay reveals *Cnksr2* deletion in *Emx*-positive excitatory neurons and *Gad2* positive inhibitory neurons increases neuronal activity in vitro

**Figure 1.**
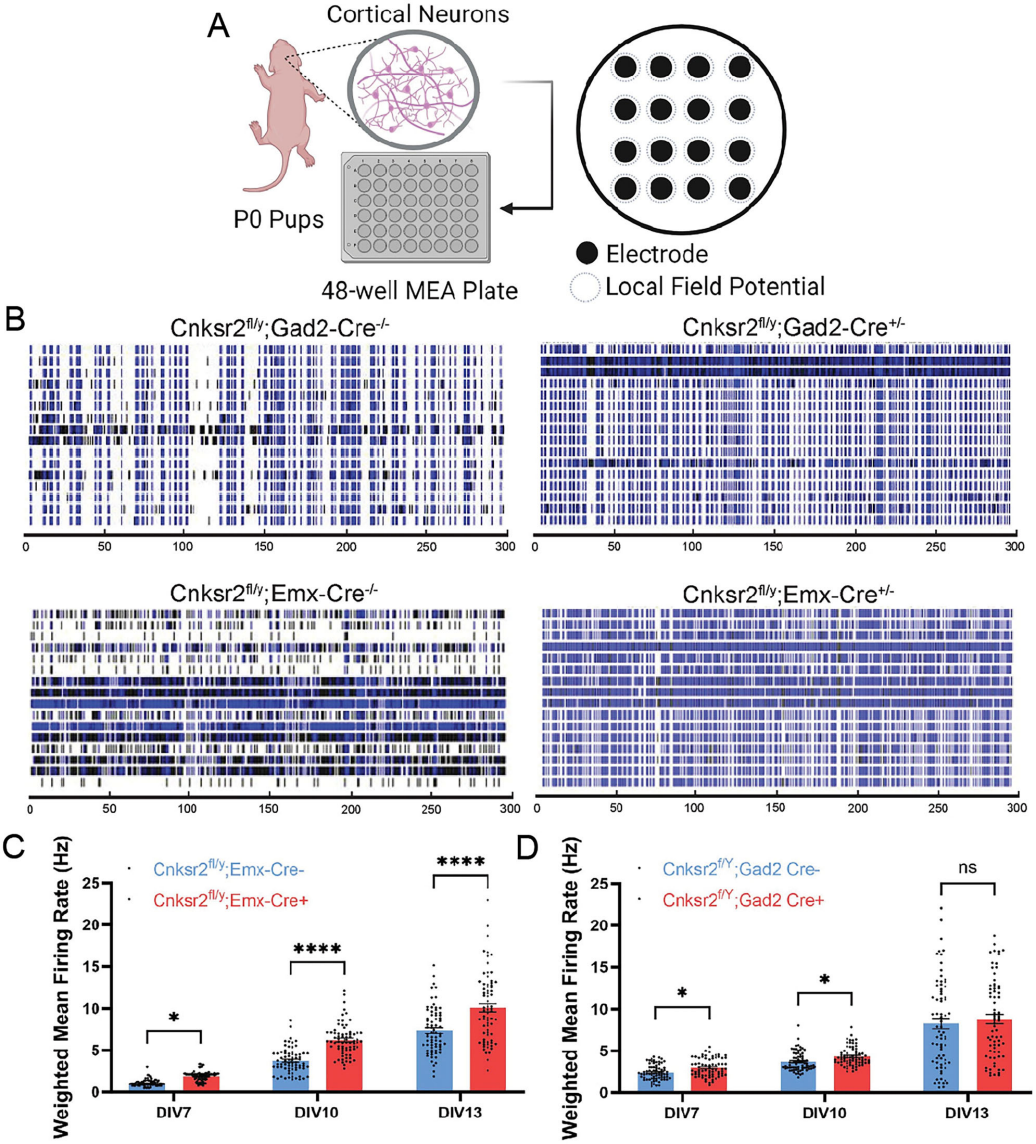
Cnksr2 loss in glutamatergic and GABAergic neurons leads to increased spontaneous firing rates of cortical neurons in vitro. ***A***, Experimental design to record spontaneous local neural activity using the multielectrode array. Cultured cortical neurons were prepared from Cnksr2^f/Y^;Emx Cre^+/−^, Cnksr2^f/Y^;Emx Cre^−/−^, Cnksr2^f/Y^;Gad2 Cre^+/−^, and Cnksr2^f/Y^;Gad2 Cre^−/−^ P0 neonatal male pups, and spontaneous neuronal activity was recorded at DIV7, 10, and 13. Three Cnksr2^f/Y^;Emx Cre^+/−^ and Cnksr2^f/Y^;Gad2 Cre^+/−^ and three Cnksr2^f/Y^;Emx Cre^−/−^ and Cnksr2^f/Y^;Gad2 Cre^−/−^ pups were used for three MEA plates. ***B***, Representative raster plots showing spontaneous activity recorded Cnksr2^f/Y^;Emx Cre^+/−^, Cnksr2^f/Y^;Emx Cre^−/−^, Cnksr2^f/Y^;Gad2 Cre^+/−^, and Cnksr2^f/Y^;Gad2 Cre^−/−^ cortical neurons on DIV 10. Electrodes 1–16 are presented on the *y*-axis, plotted against time. ***C***, Graph of spontaneous weighted mean firing rates on DIV7, DIV10, and DIV13 for Cnksr2^f/Y^;Emx Cre^+/−^ and Cnksr2^f/Y^;Emx Cre^−/−^. KO neurons displayed significantly increased weighted mean firing rates during all three time points (DIV7, *p = 0.0328; DIV10, ****p = <0.0001; DIV13, ****p < 0.0001; two-way ANOVA with multiple comparisons and repeated measures). *n = *72 wells from three plates. Data are mean ±SEM. ***D***, Graph of spontaneous weighted mean firing rates on DIV7, DIV10, and DIV13 for Cnksr2^f/Y^;Gad2 Cre^+/−^ and Cnksr2^f/Y^;Gad2 Cre^−/−^. KO neurons displayed significantly increased weighted mean firing rates on DIV7 and DIV10 (DIV7, **p = 0.000013; DIV10, ***p < 0.000001; Wilcoxon test with multiple comparisons; non-Gaussian distribution). *n = *72 wells from three plates, Data are mean ±SEM.

The diverse phenotypes resulting from the loss of Cnksr2 could reflect attributes differentially linked to specific cell types that express Cnksr2, which include both excitatory glutamatergic and GABA inhibitory neurons (Erata et al., 2021). We reasoned that discovering its cell-type–specific functions related to neuronal activity might reveal conserved or core functions as well as potentially context-specific functions. We first aimed to explore the effects of *Cnksr2* deletion in either glutamatergic or GABAergic neurons in vitro. To understand the impact of loss of Cnksr2 on neuronal activity, we recorded mean field potentials using microelectrode arrays (MEA). We conducted simultaneous local extracellular field recordings of dissociated neuronal cultures over time, allowing us to detect temporal alterations to neuronal network activity during in vitro development (Uezu et al., 2019; Erata et al., 2021). We cultured dissociated cortical neurons from *Cnksr2^fl/y^:Emx-cre^+/−^* and *Cnksr2^fl/y^:Gad2-cre^+/−^* neurons, alongside wild-type (WT) control littermates on MEA plates (Fig. 1*A*). We monitored their spontaneous activity every 3 d, from day in vitro (DIV) 7 to DIV13. Neuronal spikes were detected using a threshold-based method, which was then used for raster plot creation and further data analysis. From DIV7 onwards, both WT and Cnksr2 knock-out groups displayed synchronized neuronal firing. However, deletion of *Cnksr2* in both glutamatergic and GABAergic neurons led to an increase in the weighted mean firing rate compared with WT littermates (Fig. 1*B–D*), but with notable differences in the time course. For example, deletion of *Cnksr2* from excitatory neurons significantly affected the weighted mean firing rate at each of the measured time points (Fig. 1*C*). In contrast, deletion of *Cnksr2* from GAD2+ neurons at DIV7 and DIV10 resulted in a significant increase in weighted mean firing rate (Fig. 1*D*), which became indistinguishable from the WT group at DIV13 (Fig. 1*D*). These observations suggest that Cnksr2 has conserved functions at synapses of both glutamatergic and GABAergic neurons; however, its loss may have different network consequences during development. Given the observed effects on neural network activity, we next explored the behavioral effects of *Cnksr2* knockdown in a cell-type–specific manner in vivo.

### Conditional deletion of Cnksr2 within excitatory and inhibitory neurons demonstrate elevated levels of anxiety, loss of USVs, and increased seizures respectively in adult male mice

**Figure 2.**
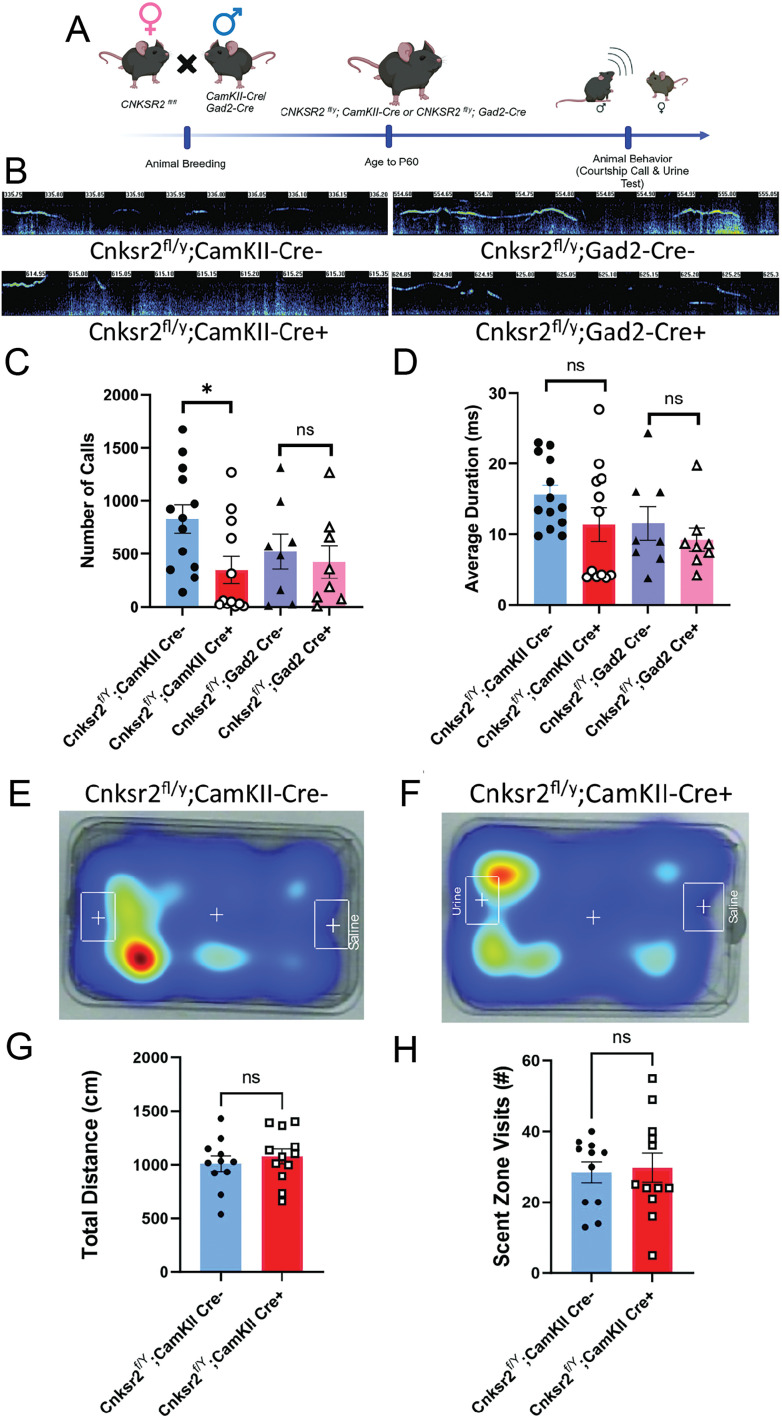
Loss of Cnksr2 in CaMKII excitatory neurons leads to USV deficits. ***A***, Schematic experimental timeline for animal breeding and behavioral analysis. ***B***, Representative spectrogram pattern of USVs in adult mice during courtship. Left, Cnksr2^f/Y^;CaMKII Cre^+/−^ and Cnksr2^f/Y^;CaMKII Cre^−/−^ spectrograms. Right, Cnksr2^f/Y^;Gad2 Cre^+/−^ and Cnksr2^f/Y^;Gad2 Cre^−/−^ spectrograms. ***C***, Left, Graphs depicting the total number of calls for each genotype in a 10 min trial. Significant differences were observed in the Cnksr2^f/Y^;CaMKII Cre^+/−^ (*n *= 12) group compared with Cnksr2^f/Y^;CaMKII Cre^−/−^ (*n *= 13). No effect of genotype was detected for the Cnksr2^f/Y^;Gad2 Cre^+/−^ (*n *= 8) group compared with Cnksr2^f/Y^;Gad2 Cre^−/−^ (*n *= 8; *p = 0.0170 and *p* = 0.6712, respectively; unpaired *t* test). ***D***, Right, Graphs depicting the average duration for each genotype. No significant difference was observed between genotypes in the duration of calls (*p* > 0.05, Mann–Whitney test). Data are ±SEM. ***E***, Representative spatial heat maps of time spent in the arena during olfactory testing. Left, Cnksr2^f/Y^;CaMKII Cre^−/−^ heat maps. ***F***, Right, Cnksr2^f/Y^;CaMKII Cre^+/−^ heat maps. The saline cassette was placed on the right side of the arena and the urine cassette on the left side of the arena. ***G***, Graph depicting the quantification of total distance traveled for each group. No significant differences were observed between genotypes (Cnksr2^f/Y^;CaMKII Cre^+/−^ *n *= 12; Cnksr2^f/Y^;CaMKII Cre^−/−^ *n *= 11; *p* > 0.05 unpaired *t* test). Data are ±SEM. ***H***, Graph depicting the sum number of scent zone visits for each group. No significant differences were observed between genotypes (Cnksr2^f/Y^;CaMKII Cre^+/−^ *n *= 12; Cnksr2^f/Y^;CaMKII Cre^−/−^ *n *= 11; *p* > 0.05 unpaired *t* test). Data are ±SEM.

**Figure 3.**
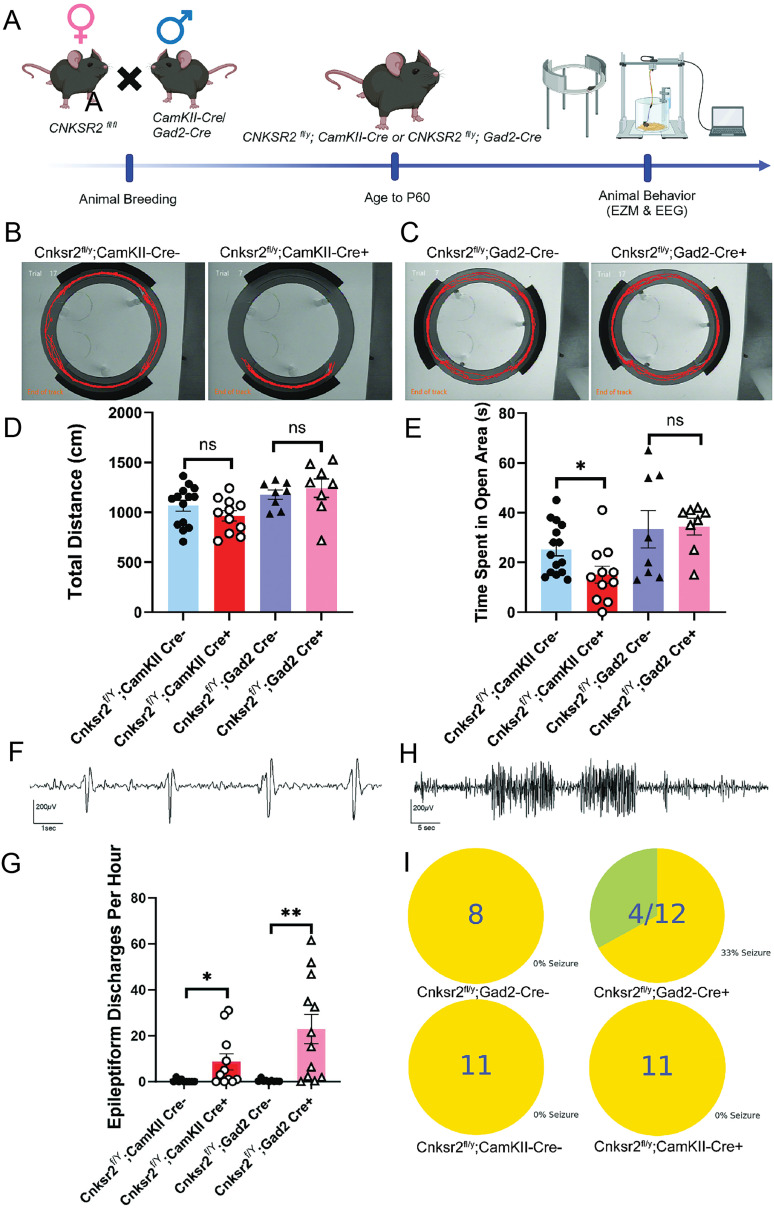
Loss of Cnksr2 in CaMKII-positive excitatory neurons contributes to elevated anxiety, while Gad2+ inhibitory deletion leads to seizures. ***A***, Schematic experimental timeline for animal breeding and behavioral analysis. ***B***, ***C***, Elevated zero maze representative locomotion traces for Cnksr2^f/Y^;CaMKII Cre^−/−^, Cnksr2^f/Y^;CaMKII Cre^+/−^, Cnksr2^f/Y^;Gad2 Cre^−/−^, and Cnksr2^f/Y^;Gad2 Cre^+/−^, respectively. ***D***, Graph of quantifications for EZM distance traveled (cm) showed no significant difference all groups (Cnksr2^f/Y^;CaMKII Cre^−/−^ *n *= 14, Cnksr2^f/Y^;CaMKII Cre^+/−^ *n *= 11; Cnksr2^f/Y^;Gad2 Cre^−/−^ *n *= 8, Cnksr2^f/Y^;Gad2 Cre^+/−^ *n *= 8; *p* > 0.05, unpaired *t* test). Data are mean ±SEM. ***E***, Graph depicting time spent in the open arms (s) of Cnksr2^f/Y^;CaMKII Cre^+/−^ (*n *= 11) showing a significant decrease compared with Cnksr2^f/Y^;CaMKII Cre^−/−^ (*n *= 14; *p = 0.0476, unpaired *t* test). Cnksr2^f/Y^;Gad2 Cre^+/−^ (*n *= 8) shows no significant change in time spent in open arms (*p* > 0.05, unpaired *t* test). Data are mean ±SEM. ***F***, Representative EEG trace of short spike–wave epileptiform discharges detected in a Cnksr2^f/Y^; Gad2 Cre^+/−^ mouse. ***G***, Graph depicting the quantification of epileptiform discharges in all groups. Both Cnksr2^f/Y^; Gad2 Cre^+/−^ (*n *= 12) and Cnksr2^f/Y^; CaMKII Cre^+/−^ (*n *= 11) displayed significantly increased number epileptiform discharges compared with their control littermates (Cnksr2^f/Y^; Gad2 Cre^−/−^ *n *= 8; Cnksr2^f/Y^; CaMKII Cre^−/−^ *n *= 11; **p = 0.0014, *p = 0.04; Mann–Whitney test). Data are mean ±SEM. ***H***, Representative EEG trace of electrographic seizures detected in a Cnksr2^f/Y^; Gad2 Cre^+/−^ mouse. ***I***, Pie chart of mice that display seizures during 1 d of recording. Cnksr2^f/Y^;CamKII Cre^−/−^, Cnksr2^f/Y^;CamKII Cre^+/−^, and Cnksr2^f/Y^;Gad2 Cre^−/−^ lack seizure activity but 4/12 Cnksr2^f/Y^;Gad2 Cre^+/−^ mice exhibit seizures with a total of 36 events collectively among the 4 mice. Data are mean ± SEM.

A critical question in the field of epilepsy–aphasia revolves around the origin of the complex phenotypes and their relationship to each other. For example, it is currently unclear whether the seizures experienced by patients are the cause of their language loss (Unterberger et al., 2021). Elevated neuronal excitation and seizures are often comorbid in neurologic disorders that present with behavioral abnormalities (Rubenstein and Merzenich, 2003). To address this, we explored the conditional deletion of *Cnksr2* in glutamatergic and GABAergic neurons separately. For the deletion of *Cnksr2* in excitatory cells, we crossed *Cnksr2^fl/fl^* females with *CaMKII-cre^+/−^*males. This resulted in male offspring being *Cnksr2^fl/y^:CaMKII-cre^+/−^*. A similar breeding approach of crossing *Cnksr2^fl/fl^* females with *Gad2-cre^+/−^*males was used. These breeding schemes were crucial to ensure littermate controls from the same cross (Figs. 2*A*, 3*A*). Following genotyping, the mice were aged to P60 and then subjected to behavioral assays for USVs, olfactory discrimination test, elevated zero maze (EZM), and seizure analyses. To analyze courtship-related vocalizations, sonograms were recorded for males of both genotypes in response to a female stimulus (Fig. 2*B*, left panels, *CaMKII-cre*; right panels, *Gad2-cre*)*.* Adult male mice typically produce ultrasonic vocalizations at 30 kHz to court females for reproduction (Whitney, 1970). Quantification of these USV revealed that when compared with littermate controls, *Cnksr2^fl/y^:CaMKII-cre^+/−^* mice exhibited a decrease in the number of calls (*Cnksr2^f/Y^;CaMKII Cre−*, 828.538 calls; *Cnksr2^f/Y^;CaMKII Cre+*, 346.917 calls, *p* = 0.0170) whereas there was no effect of genotype for *Cnksr2^fl/y^:Gad2-cre^+/−^* mice (Fig. 2*C*). Neither line of mice exhibited differences in the total duration of existing calls (Fig. 2*D*). Since olfaction is a crucial component of social behaviors in mice (Egnor and Seagraves, 2016), we also analyzed the olfactory responses in *Cnksr2^fl/y^:CaMKII-cre^+/−^* and control mice, since CaMKII-cre can also be expressed in the olfactory bulb (Zou et al., 2002). We used an olfactory discrimination test in which mice were presented with a female urine cassette and a saline cassette within the same cage (Fig. 2*E,F*). The number of mice spending more time in the scent zone versus the saline zone was similar (7/11 WT and 9/12 *Cnksr2^fl/y^:CaMKII-cre^+/−^*). There was no difference between genotypes in the total distance traveled in the arena (Fig. 2*G*). Likewise, there was also no effect of genotype on the number of visits to the urine scent zone (Fig. 2*H*). These data suggest that *Cnksr2^f/Y^;CaMKII Cre−* and *Cnksr2^f/Y^;CaMKII Cre+* males do not exhibit differences in responses to olfactory stimuli; thus, the reduced USVs are likely not due to differences in olfaction. To test for genotype effects on anxiety, EZM analysis was also conducted. These results revealed that *Cnksr2^fl/y^:CaMKII-cre^+/−^* mice displayed elevated levels of anxiety, spending less time in the open arms of the maze (Fig. 3*B,E*). In contrast, *Cnksr2^fl/y^:Gad2-cre^+/−^* mice showed no significant differences between control and experimental groups (Fig. 3*C,E*). Importantly, neither group exhibited differences in total distances traveled (Fig. 3*D*). Finally, we also tested whether the loss of Cnksr2 in excitatory or inhibitory neurons modulated epileptiform activity and seizures. Twenty-four hour EEG recordings were analyzed for epileptiform discharges (Fig. 3*F*; representative trace from *Cnksr2^fl/y^:Gad2-cre^+/−^*). *Cnksr2^fl/y^:CaMKII-cre^+/−^* mice had an increase in epileptiform discharges when compared with wild-type controls (Fig. 3*G*). *Cnksr2^fl/y^:Gad2-cre^+/−^* mice also exhibited elevated epileptiform discharges, although to a greater extent (Fig. 3*G*). EEG recordings were also analyzed for electrographic seizures (Fig. 3*H*, representative trace from *Cnksr2^fl/y^:Gad2-cre^+/−^*). No seizures were detected for the wild-type littermates of either genotype or in the *Cnksr2^fl/y^:CaMKII-cre^+/−^* mice (Fig. 3*I*). In contrast, 4 out of 12 *Cnksr2^fl/y^:Gad2-cre^+/−^* mice exhibited electrographic seizures during the recording period, with a total of 36 events in these animals (Fig. 3*I*, top right panel). Overall, these data demonstrated elevated neuronal activity following loss of Cnksr2 from either CaMKII-cre+ or Gad2-cre+ neurons in vivo, consistent with our MEA data, but the effect may be stronger in Gad2-cre mice, leading to a trend of increased seizures. In contrast, altered vocal communication via USVs specifically arises downstream of the loss of Cnksr2 in CaMKII-cre+ cells.

### Deletion of *Cnksr2* in *Emx1* positive neurons recapitulates loss of ultrasonic vocalization and increased levels of anxiety in adult male mice

**Figure 4.**
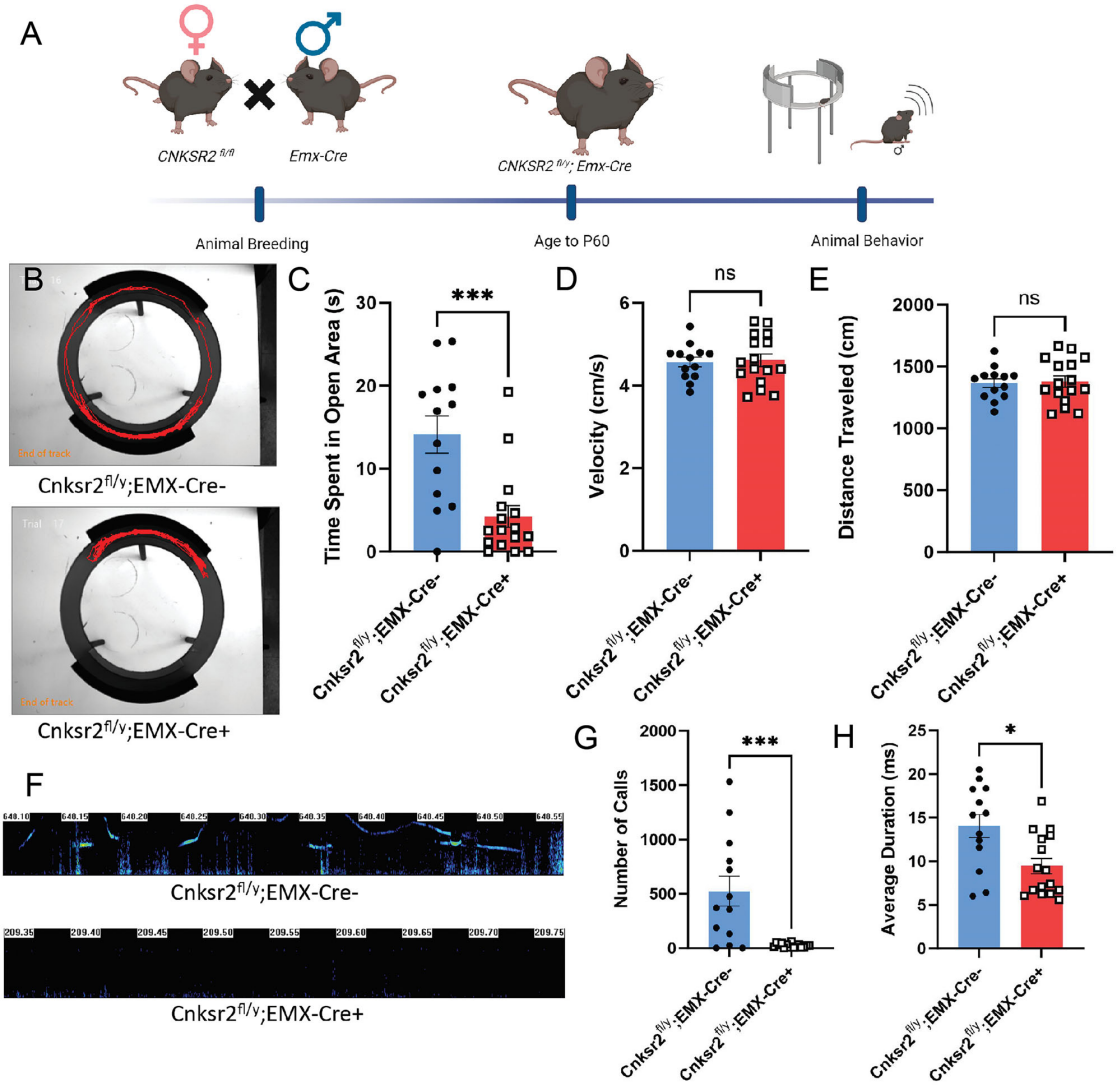
Loss of Cnksr2 in Emx^+^ glutamatergic neurons recapitulates the elevated anxiety and reduced USV phenotypes of knock-out mice. ***A***, Schematic experimental timeline for animal breeding and behavioral analysis. ***B***, Representative locomotion traces during elevated zero maze for Cnksr2^f/Y^;Emx Cre^−/−^ (top) and Cnksr2^f/Y^;Emx Cre^+/−^ (bottom) male mice. ***C***, Graph depicting the total time spent in the open arms of the maze for both groups is shown. Cnksr2^f/Y^;Emx Cre^+/−^ (*n *= 16) mice spent significantly less time in the open arms than the Cnksr2^f/Y^;Emx Cre^−/−^ (*n *= 13; ***p = 0.0009, Mann–Whitney test). Data are mean ± SEM. ***D***, Graph of the locomotor velocity. No significant effect of genotype in either group was detected (*p* > 0.05; unpaired *t* test). Cnksr2^f/Y^;Emx Cre^+/−^ (*n *= 16) and Cnksr2^f/Y^;Emx Cre^−/−^ (*n *= 13). Data are mean ± SEM. ***E***, Graph of the distance traveled. No significant effect of genotype in either group was detected (*p* > 0.05; unpaired *t* test). Cnksr2^f/Y^;Emx Cre^+/−^ (*n *= 16) and Cnksr2^f/Y^;Emx Cre^−/−^ (*n *= 13). Data are mean ± SEM. ***F***, Representative spectrogram pattern of USVs in adult mice during courtship. Top, Cnksr2^f/Y^;Emx Cre^−/−^ spectrograms. Bottom, Cnksr2^f/Y^;Emx Cre^+/−^ spectrograms. ***G***, Graph depicting the total number of calls produced by male mice exposed to females. Cnksr2^f/Y^;Emx Cre^+/−^ mice (*n *= 16) had a significant decrease in USVs produced compared with Cnksr2^f/Y^;Emx Cre^−/−^ (*n *= 13; ***p = 0.0004, unpaired *t* test). Data are mean ± SEM. ***H***, Graph showing the average duration of calls produced by male mice when exposed to female mice. Cnksr2^f/Y^;Emx Cre^+/−^ mice (*n *= 16) had a significant decrease in the average duration of USVs compared with Cnksr2^f/Y^;Emx Cre^−/−^ (*n *= 13) male mice (*p = 0.0196, Mann–Whitney test). Data are mean ± SEM.

The above results suggested that loss of Cnksr2 from excitatory neurons may specifically lead to the USV phenotypes of these mice; however, it is known that CaMKII promoter-based strategies can result in sparse expression in some inhibitory neuron subtypes (Keaveney et al., 2020). Thus, to confirm whether loss of *Cnksr2* from glutamatergic neurons is causative for the USV phenotypes, we used empty spiracles homeobox (*Emx)-Cre*. Emx expression is limited to the cortex, hippocampus, and olfactory bulbs (Simeone et al., 1992; Gorski et al., 2002). Emx-cre results in the deletion of floxed alleles in redial glia, resulting in cre-mediated recombination in both glutamatergic neurons and some glia. However, *Cnksr2* shows minimal expression in glial cells, including astrocytes (Zhang et al., 2014), and glial cells are not targeted by CaMKII-cre. For this analysis, we generated male mice with Cnksr2 knocked out in *Emx*-positive glutamatergic neurons (*Cnksr2^fl/y^:Emx-cre^+/−^* ;Fig. 4*A*). We allowed these mice to age to postnatal day 60 before conducting behavioral testing to assess anxiety and vocal communication behaviors. Analysis of the EZM test (Fig. 4*B*) revealed that the knock-out mice showed a significant reduction in time spent in the open arms compared with wild-type littermates (Fig. 4*C*). To differentiate between anxiety and motor deficits, we also measured the velocity (Fig. 4*D*) and distance traveled (Fig. 4*E*) by these mice, finding no differences between genotypes. Thus, the effects on time spent in the open arms of the maze suggested that deleting *Cnksr2* in glutamatergic cells of the cortex and hippocampus leads to elevated anxiety. We then examined USV production by recording sonograms during courtship behavior (Fig. 4*F*), as in the above CaMKII-cre and Gad-2-cre analysis. Analysis revealed the conditional knock-out male mice showed a significant reduction in both the number (Fig. 4*G*) and duration (Fig. 4*H*) of USVs produced. These data collectively confirm that deletion of *Cnksr2* in excitatory neurons of the cortex and hippocampus results in increased anxiety levels and a reduction in USV production in male mice.

### Brain regional specific deletion of Cnksr2 within CaMKII-positive neurons in the anterior cingulate cortex leads to loss of ultrasonic vocalization and does not affect anxiety

**Figure 5.**
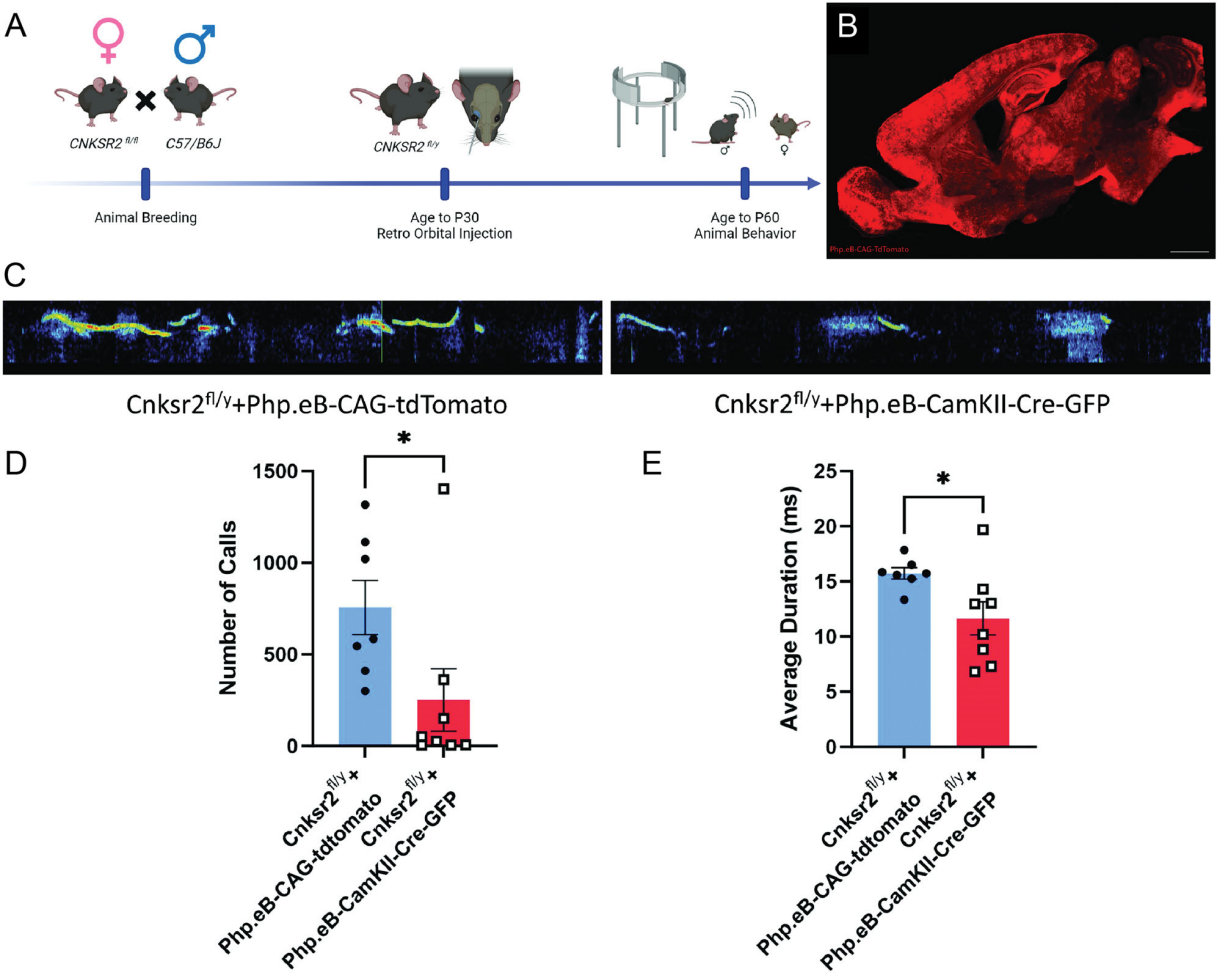
Retro-orbital viral-mediated knock-out of Cnksr2 affects USV production. ***A***, Schematic experimental timeline for animal breeding, injections, and behavioral analysis. ***B***, Representative histology image for retro-orbital viral infection with Php.eB-CAG-tdTomato in a sagittal section of 50 µm at P60. Php.eB-CAG-tdTomato expression can be seen throughout the entire brain, particularly in the cortex. Scale bar, 500 µm. ***C***, Representative spectrogram pattern of USVs in adult mice during courtship. Left, Cnksr2^f/Y^-AAV-Php.eB-CAG-tdTomato. Right, Cnksr2^f/Y^-AAV-Php.eB-CaMKII-Cre-GFP. ***D***, The number of calls was significantly decreased in Cnksr2^f/Y^-AAV-Php.eB-CaMKII-Cre-GFP group (*n = *8) when compared with Cnksr2^f/Y^-AAV-Php.eB-CAG-tdTomato (*n = *7; *p = 0.0200, Mann–Whitney test). Data are mean ± SEM. ***E***, Average duration of calls was significantly decreased in Cnksr2^f/Y^-AAV-Php.eB-CaMKII-Cre-GFP group (*n = *8) when compared with Cnksr2^f/Y^-AAV-Php.eB-CAG-tdTomato (*n = *7; *p = 0.0312 unpaired *t* test). Data are mean ± SEM.

Vocal communicative disorders are complex, and their etiology in most cases remains enigmatic. The finding that USV abnormalities downstream of loss of Cnksr2 track to its functions in excitatory neurons provides a possible opportunity to further narrow down the brain regions responsible for this phenotype. An attractive approach to do so could leverage viral-mediated approaches to delete Cnksr2 in specific brain regions using the CaMKII promoter. As a prelude to brain regional deletion of Cnksr2, we first sought to validate that the overall approach could recapitulate the USV phenotypes observed in the EMX and CaMKII-cre genetic lines. To do so, we utilized retro-orbital delivery of AAV-PhP.eB-CAG-tdTomato (control) and AAV-PhP.eB-CaMKII-Cre-GFP (experimental) virus into *Cnksr2^fl/y^* mice at postnatal day 21 (Fig. 5*A*). Viral infection using this approach was validated by direct visualization of tdTomato to verify brain-wide transduction under these conditions (Fig. 5*B*). We next assessed USVs on postnatal day 60. USVs were again detected using sonograms (Fig. 5*C*). Analysis from male mice of both viral conditions showed a significant decrease in both the quantity (Fig. 5*D*) and duration (Fig. 5*E*) of calls produced by mice transduced with CaMKII-Cre when compared with those transduced with tdTomato-expressing AAV. These results suggest that deletion of *Cnksr2* using viral expression of Cre by the CaMKII promoter can indeed recapitulate the USV deficits of conditional knock-out mice. These results supported further investigations to determine Cnksr2's role in specific cortical regions regarding USVs.

**Figure 6.**
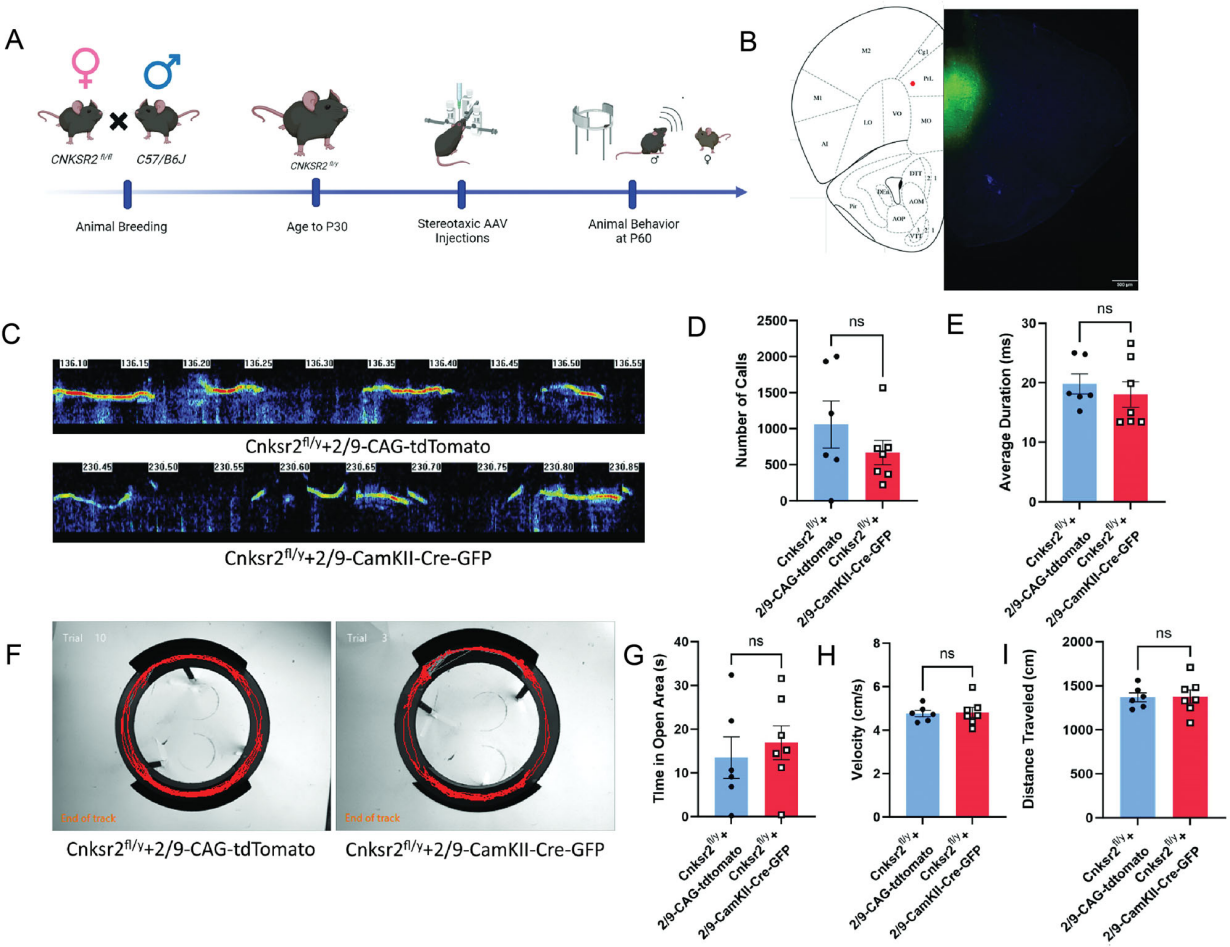
Deletion of Cnksr2 within CaMKII-positive glutamatergic neurons of the mPFC does not affect USV production. ***A***, Schematic experimental timeline for animal breeding, injections, and behavioral analysis. ***B***, Representative histology image for mPFC viral targeting of Cnksr2 in coronal section of 50 µm at P60. AAV2/9-*CamKII-Cre-GFP* expression can be seen in the mPFC. Scale bar, 500 µm. ***C***, Example spectrogram pattern of USV's in (top) Cnksr2^f/Y^-AAV2/9-CAG-tdTomato and (bottom) Cnksr2^f/Y^-AAV2/9-CaMKII-Cre-GFP adult male mice during courtship. Graphs of (***D***) total number of calls and (***E***) average call duration. No significant effect was observed on the number of calls and average duration of calls (*p* > 0.05 unpaired *t* test; *n *= 6). Data are mean ± SEM. ***F***, Representative locomotion traces in the elevated zero maze for (left) Cnksr2^f/Y^-AAV2/9-CAG-tdTomato and (right) Cnksr2^f/Y^-AAV2/9-CaMKII-Cre-GFP male mice. Graphs showing elevated zero maze data for (***G***) time in the open arms; (***H***) average velocity; and (***I***) total distance moved. No significant effect of treatment was observed in any of these metrics (*p* > 0.05; unpaired *t* test; Cnksr2^f/Y^-AAV2/9-CAG-tdTomato *n *= 6 and Cnksr2^f/Y^-AAV2/9-CaMKII-Cre-GFP *n *= 7). Data are mean ± SEM.

**Figure 7.**
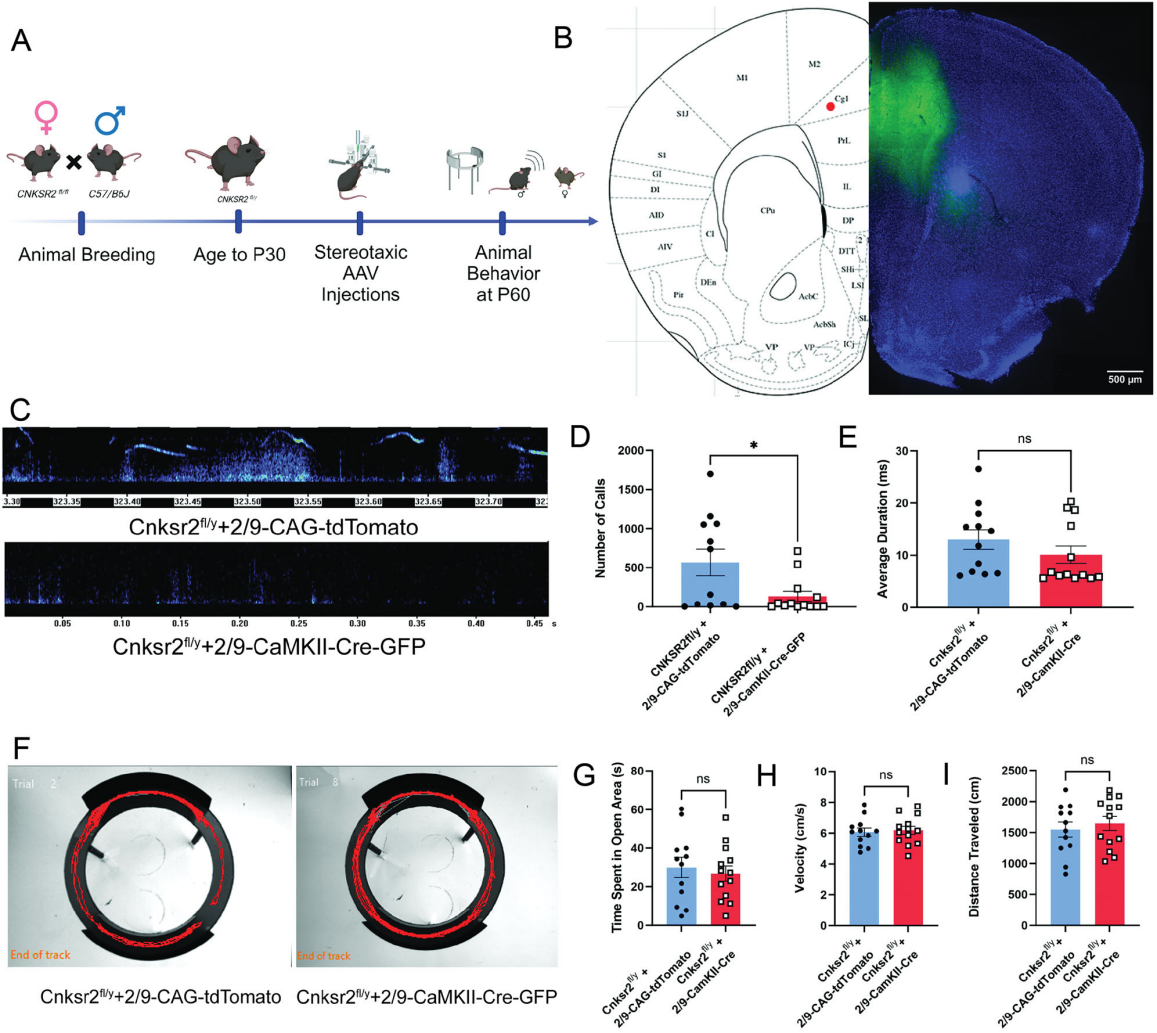
Deletion of Cnksr2 within CaMKII-positive glutamatergic neurons of the ACC leads to altered USV behavior. ***A***, Schematic of experimental timeline for animal breeding, injections, and behavioral analysis. ***B***, Representative histology image for ACC viral targeting of Cnksr2 in coronal section of 50 µm at P60. *AAV2/9-CamKII-Cre-GFP* expression can be seen in the ACC. Scale bar, 500 µm. ***C***, Example spectrogram pattern of USV's in (top) Cnksr2^f/Y^-AAV2/9-CAG-tdTomato and (bottom) Cnksr2^f/Y^-AAV2/9-CaMKII-Cre-GFP adult male mice during courtship. Graphs of (***D***) total number of calls and (***E***) average call duration. The number of calls was significantly decreased in Cnksr2^f/Y^-AAV2/9-CaMKII-Cre-GFP (*n = *13) when compared with Cnksr2^f/Y^-AAV2/9-CAG-tdTomato groups (*n = *12). The average duration was not affected (*p* > 0.05, Mann–Whitney test; number of calls **p = 0.0089, unpaired *t* test). ***F***, Elevated zero maze representative locomotion traces shown for (left) AAV2/9-CAG-tdTomato and (right) AAV2/9-CaMKII-Cre-GFP injections. Graphs showing elevated zero maze data for (***G***) time in the open arms; (***H***) average velocity; and (***I***) total distance moved. No significant effect of treatment was observed in any of these metrics (*p* > 0.05; unpaired *t* test; *n = *13; *p* > 0.05; unpaired *t* test). Data are mean ± SEM.

At least two regions of the cortex, the medial prefrontal cortex (mPFC) and anterior cingulate cortex (ACC), have been implicated in vocal communication in mice and rats (Bennett et al., 2019; Gan-Or and London, 2023). Both regions have projections to the midbrain region of the PAG, the vocal gating center, which is known to regulate USVs. Thus, we hypothesized that Cnksr2 function in either of these two regions—mPFC or ACC—is necessary for normal USV production. To test this hypothesis, we used a stereotaxic approach to conditionally delete *Cnksr2* within *CaMKII*-positive cells in the mPFC at postnatal day 30 and then assessed USVs postnatal day 60 (Fig. 6*A*). Analysis revealed no significant impact on USV calls or duration when *Cnksr2* was deleted in *CaMKII*-positive cells within the mPFC (Fig. 6*C,D*). Analysis of anxiety in the mPFC CaMKII-cre–expressing mice also revealed no effect of viral condition on time in open arms, velocity, or distance traveled (Fig. 6*E,F*). Next, the role of Cnksr2 was also similarly tested in the ACC (Fig. 7*A,B*). Quantification of USV metrics revealed a significant reduction in the number of calls; however, of the calls remaining, there was no effect of duration (Fig. 7*D*). Interestingly, loss of Cnksr2 in excitatory neurons using either CaMKII-cre or EMX-cre resulted in elevated anxiety. This elevated anxiety could underlie the comorbid impaired USVs. Analysis of EZM data revealed that despite the significant effect on USV calls, there was no effect of viral condition on the time in the open arms, and velocity of travel or distance traveled was unaffected (Fig. 7*E,F*). These results suggest that Cnksr2 in glutamatergic cells of the ACC plays a role in the initiation of USVs in adult male mice. It also reveals that the USV phenotypes upon loss of Cnksr2 are independent of the elevated anxiety in these mice. The specific involvement of Cnksr2 in the ACC, as opposed to the mPFC, highlights a more precise role for this region in vocal communication, particularly in the context of Cnksr2's function.

**Figure 8.**
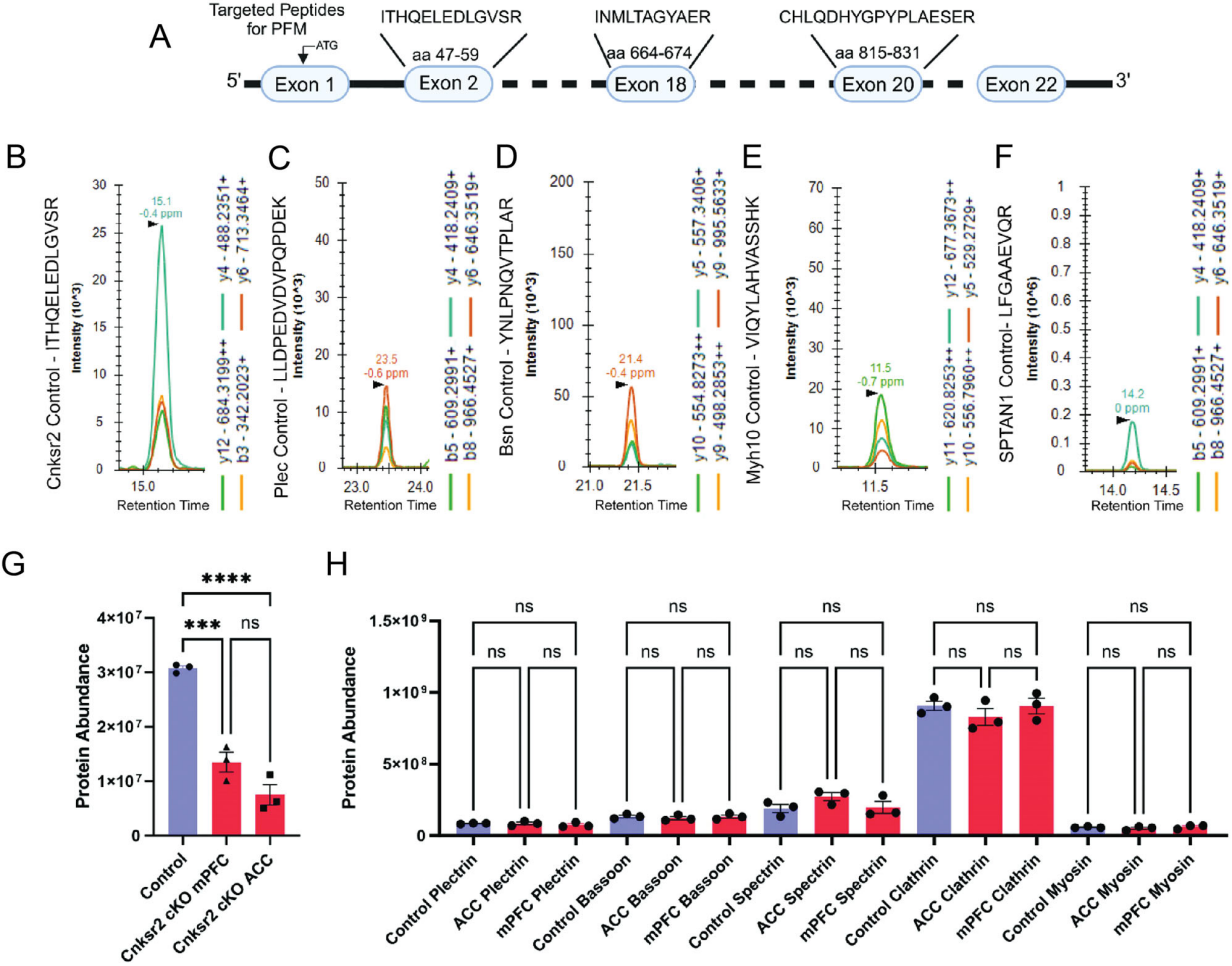
Quantitative analysis of the viral-mediated depletion of Cnksr2 from the ACC and mPFC. ***A***, Schematic showing three different peptides and the exons they are derived from that were targeted for PRM LC-MS assay to quantify relative protein expression levels across WT and cKO animals. ***B–F***, Representative PRM chromatograms of the examples for the most abundant product ions for (***B***) Cnksr2, (***C***) plectin, (***D***) bassoon, (***E***) myosin 10, and (***F***) alpha II spectrin. ***G***, Quantification of protein abundance of Cnksr2 in control and cKO ACC and mPFC regions as calculated by averaging the abundance of all three peptides targeted for all samples. Cnskr2 was significantly depleted in the cKOs compared with the WT control (****p = <0.0001 for ACC cKO, ***p = 0.0004 for mPFC cKO, one-way ANOVA with multiple comparisons). *n *= 3 animals per group. Data are mean ± SEM. ***H***, Quantification of protein abundance for control proteins in WT and cKO ACC and mPFC regions as calculated by averaging the abundance of all three peptides targeted for all samples. No significant differences were observed. Data are mean ± SEM.

We next aimed to verify the efficiency of viral-mediated Cnksr2 depletion to confirm the different behavioral results observed were not attributable to differences in the depletion levels between the ACC and mPFC (Fig. 8). To do so, we conducted targeted parallel reaction monitoring (PRM) after transduction with Cre or control virus with three peptides from each protein to quantify the levels of Cnksr2 (Fig. 8*A*) versus five unrelated control proteins [plectin, bassoon, alpha II spectrin (SPTAN1), clathrin, and myosin 10; representative peptide chromatograms; Fig. 8*B*–*F*]. Microdissected ACC and mPFC exhibited significant and comparable decreases in Cnksr2 protein levels compared with tissue without cre (Fig. 8*G*). In contrast, there were no significant differences for the unrelated control proteins (Fig. 8*H*). Thus, the different behavioral effects of Cnksr2 depletion between these brain regions are likely a reflection of its brain regional roles.

## Discussion

Epilepsy–aphasia syndrome (EAS) is a complex disorder with a poorly understood genetic and cellular basis. Previous research indicates that mutations in Cnksr2 linked to neurodevelopmental disorders, including EAS, involve significant deletions and nonsense mutations, resulting in a loss of function (Houge et al., 2012). We previously reported a mouse model with loss of Cnksr2 following germline deletion of a floxed exon 2. These mice lack Cnksr2 protein and modeled several phenotypes analogous to those of patients, including seizures and the progressive loss of vocal communication (USVs). Given the paucity of mechanistic studies at the genetic and cellular level for EAS, we took advantage of the conditional allele for Cnksr2 to discover and disentangle its roles in the neuronal cell populations it is expressed in, excitatory and inhibitory neurons. Our results highlight the core as cellular and behavioral functions of Cnksr2, which is important for understanding the interplay between comorbidities of EAS.

Our multielectrode array recordings highlight that Cnksr2 plays an important role in regulating neuronal network activity. Loss of Cnksr2 resulted in elevated neuronal activity, whether it was deleted from excitatory or inhibitory neurons, which was surprising given the opposing roles of these neuronal cell types. While it is currently unclear how the loss of Cnksr2 in both neuronal populations leads to elevated network activity, we speculate it is likely by distinct mechanisms. We and others have previously shown that loss of Cnksr2 in excitatory neurons leads to a decrease in spine density (Lim et al., 2014). Interestingly, we previously showed that loss of the dendritic spine Arp2/3 complex subunit, ArpC3, also led to reduced spine density and a paradoxical increase in neuronal activity (Kim et al., 2015). In the case of ArpC3 loss, we found that excitatory synapses shifted from axospine contacts to axodendritic shaft synapses. We previously speculated that the increase in spontaneous firing might be due to the loss of the electrical insulator function of spines (Harnett et al., 2012; Tønnesen et al., 2014). Given the similar results for Cnksr2, future work will be needed to determine if this might be a mechanism at play upon its loss. Loss of *Cnksr2* in inhibitory neurons also resulted in increased activity. The most parsimonious explanation is that loss of Cnksr2 in GABAergic neurons may reduce their excitatory input, leading to reduced inhibition of excitatory neurons. These mechanisms remain speculative, however.

Acquired aphasia is one of the most severe deficits seen in EAS disorders, and its genetic and cellular basis is poorly understood. It is a highly penetrant phenotype of Cnksr2 variants in humans. A recent review of case reports of male patients with Cnkrs2 variants found that 31 out of 33 have language difficulties (Ito and Nagata, 2022). Of these, all but one was reported to have seizures. This fits with the broader literature on EAS, in which aphasia coincides with the occurrence and worsening of seizures (Bonardi et al., 2020). Given that loss of speech and/or language can have devastating impacts on patients, understanding the mechanisms underlying aphasia is critical. While mouse USVs are extensively used in studies to model human vocal communication and associated disorders (Fischer and Hammerschmidt, 2011; Chabout et al., 2016; Wang et al., 2016), it is important to acknowledge limitations that may hinder the translation of results from mice to human conditions. While human language is learned, mouse USVs are innate (Whitney, 1970). Additionally, most EAS patients retain the ability to laugh and cry, which are innate vocalizations. Despite this, corticostriatal and corticobulbar circuits similar to those that control learned vocalizations in humans are also implicated in USV production in mice, suggesting some overlap exists between the two species (Arriaga et al., 2012).

Here, we report that loss of Cnksr2 from cortical excitatory neurons, using two different Cre lines, is sufficient to significantly impair ultrasonic vocalizations in adult male mice, providing some of the first insights into its cellular basis. There has been debate about the role of the cortex in regulating USVs in mice. The evidence is conflicting: some studies have shown that mice can produce USVs even after complete removal of the cortex, while others have successfully induced USV production by directly stimulating specific cortical areas (Hammerschmidt et al., 2015; Okobi et al., 2019). Our results fit with the notion that cortical regions normally play a modulatory role in USVs and that complete removal of the cortex results in plasticity in other brain regions as a compensatory mechanism. We further demonstrate that the effects of Cnksr2 on USVs are likely mediated by the loss of Cnkrs2 from the anterior cingulate cortex (ACC). Importantly, the elevated anxiety phenotype that was cosegregated with the loss of Cnksr2 in cortical glutamatergic neurons did not localize to the ACC, supporting the notion that anxiety is not a primary cause of the impaired USVs in these mice. Interestingly, the ACC in humans is involved in the control of emotional vocal utterances and other paralinguistic features of speech (Arriaga and Jarvis, 2013; Nieder and Mooney, 2020). Moreover, it has been established that the PAG acts as the vocal gating center (Jürgens and Pratt, 1979) and that neurons residing in the ACC project to this region of the brain (Lee et al., 2022). Future work to manipulate Cnksr2 within this ACC to PAG projection is needed to determine whether it will affect USV production.

In conclusion, this study provides evidence that aphasia and seizures are governed by glutamatergic and GABAergic neurons, respectively, in the Cnksr2 model of EAS. These results are the first to our knowledge to highlight the cellular etiology underlying these complex phenotypes in an EAS model organism. This provides new insights into the possible mechanisms underlying EAS in humans and suggests the ACC is a focal point for the role of Cnksr2 in regulating vocal communication. Based on these results, it will be interesting to determine if other EAS variants, such as those reported for *GRIN2A*, also mediate their effects in a similar cell and brain region–specific manner.

## Synthesis

Reviewing Editor: Niraj Desai, National Institute of Neurological Disorders and Stroke Decisions are customarily a result of the Reviewing Editor and the peer reviewers coming together and discussing their recommendations until a consensus is reached. When revisions are invited, a fact-based synthesis statement explaining their decision and outlining what is needed to prepare a revision will be listed below. The following reviewer(s) agreed to reveal their identity: NONE. Note: If this manuscript was transferred from JNeurosci and a decision was made to accept the manuscript without peer review, a brief statement to this effect will instead be what is listed below.

On the basis of the authors' responses to the original reviews and my own reading, I think this manuscript deserves publication in eNeuro.
